# Surgical reconstruction of the acromioclavicular joint: Can we identify the optimal approach?

**DOI:** 10.1007/s11751-018-0314-1

**Published:** 2018-07-05

**Authors:** Alexander S. North, Tracey Wilkinson

**Affiliations:** University of Dundee, Dundee, UK

**Keywords:** Acromioclavicular, Rockwood, Reconstruction, Anatomical, Hook plate, Biomechanics

## Abstract

Injuries to the acromioclavicular (AC) joint are common, tending to occur secondary to traumatic injuries. Rockwood grade IV, V and VI injuries involve complete dislocation of the joint and require surgical reconstruction, with inconclusive literature on whether grade III injuries should be surgically or conservatively managed. There are over one hundred reported surgical techniques which reconstruct the AC joint, with little indication of which methods achieve the best results. Techniques can generally be considered as: anatomical reduction; CC ligament reconstruction; and anatomical reconstruction. Techniques which implant hardware to reduce the AC joint, such as the hook plate, are commonly implemented, but have been shown to alter the mechanics of the joint significantly, resulting in poor short-term and long-term outcomes. Methods which reconstruct both the acromioclavicular and coracoclavicular ligaments are comparatively new, and early reports suggest that they achieve biomechanical properties similar to the native joint. More focus should be placed on such techniques in the future to determine whether they offer a more suitable approach to improve patient outcomes following AC joint reconstruction.

## Background

Surgical fixation of the acromioclavicular (AC) joint dates back to Cooper (1861) [1], who reduced and fixated the joint with silver wire. A similar approach using K-wires was still implemented 100 years later [2], with the aim of reducing the AC joint anatomically and allowing healing of the surrounding soft tissues. K-wire fixation has become unpopular due to its disruption of the articular surfaces, thus accelerating osteoarthritic changes [2]. Currently there are over 100 techniques of surgical fixation of the AC joint described in the literature. There are some discrepancies between approaches for acute and chronic injuries, but there is little consensus as to which methods are the most effective.

More recent techniques have moved away from fixation using implanted hardware, but one of the few techniques still implemented are hook plates [3]. These are implanted inferiorly to the acromion with screws securing the implant to the superior surface of the clavicle. Hook plates remain in situ for up to 6 months to allow ligamentous healing [3].

Weaver and Dunn’s seminal paper was the first to describe a technique of ligamentous reconstruction, negating the need for hardware implementation and removal [4]. Although the Weaver and Dunn technique is only utilised for chronic AC joint pathology, it inspired multiple techniques of CC ligament reconstruction implemented for acute injuries [4–6]. Alternatively, some authors favour simultaneous reconstruction of the AC and CC ligaments [7]. This essentially creates three categories of surgical approaches for acute AC joint injuries: anatomical reduction, CC ligament reconstruction and anatomical reconstruction.

However, despite it being widely accepted that high-grade AC injuries should be managed surgically, there have been limited attempts to identify which approach achieves the best clinical and/or biomechanical outcomes.

## Aim

To compare the three general categories of reconstructive approaches in an attempt to provide some insight into the optimal surgical management of AC joint injuries.

### Surgical management of AC joint injuries

It would seem reasonable that the merits of a surgical approach could be assessed on the similarity of the result to the native complex. However, another appropriate aim would be to achieve good subjective outcomes. Much of the literature is based on the restoration of normal anatomy [8], and evidence suggests that failure to recreate an anatomically ‘normal’ joint results in instability and poorer clinical outcomes [9, 10].

McConnell et al. [11] reported that the rigidity of the hook plate reconstruction was not significantly different from the native complex, but the authors only tested displacement along a superior vector. Nüchtern et al. [12] confirmed stability in the superior direction and also for axial rotation, but found significantly increased residual motion in the horizontal plane in comparison with the normal joint. Testing was extensive in Nüchtern et al’s paper [12], as they assessed the cadaveric specimens immediately post-construction and following 1000 repetitions of cyclical loading, which they hypothesised replicated post-operative physiotherapy. However, of their six hook plate reconstructions, two failed during cyclical loading and were omitted from results. Perhaps this is indicative of a poor implant option, but may also be due to lower bone density in elderly subjects, or poor surgical technique.

Clinical outcomes for hook plate reduction are variable. McKee et al. [3] reported that only 81% (30/37) maintained reduction at 1 year, but did not outline their criteria for what was considered as maintained reduction. Di Francesco et al. [13] demonstrated, through MRI evaluation, that 88% (37/42) had complete CC ligament healing at 1 year, thus maintaining reduction. These authors noted an association between the 12% (5/42) who had residual instability and poor clinical outcome. Yoo et al. [14] found a 6% mean increase in CC distance from immediate post-operative status. All were completely reduced post-operatively, and none experienced recurrent subluxation (defined as a CC distance of ≥ 150%). McKee et al. [3] and Yoo et al. [14] both reported good clinical outcomes using the Constant score, but neither implemented the test to allow comparison with the uninjured shoulder. However, some authors report unusually high infection rates [15] and a high incidence of acromial osteolysis, in which the plate penetrates the cortex of the acromion [14].

The modified Weaver–Dunn procedure is now reserved for chronic AC joint pathology in which the CC ligament is unlikely to heal. The procedure was suboptimal in reconstructing the CC ligaments for two reasons. First, the vector of the grafted coracoacromial ligament results in considerably different moment generation and an ultimate strength of approximately 20% of the native CC ligaments [16]. Second, compression of the AC joint improves stability and thus distal clavicle excision contributes to anteroposterior instability [17]. However, the technique led to the development of further techniques which focus on an anatomical reconstruction of the CC ligaments following an acute injury, including: TightRope repair [5], two-bundle repair [6] and tendon graft reconstruction [14].

Only three papers have investigated CC reconstruction using cadavers: Costic et al. [8], Mazzocca et al. [18] and Deshmukh et al. [19] (Table 1). Mazzocca et al. [18] reported that CC reconstruction achieved biomechanical results equivalent to the native complex, but others have found inferior stability [8, 19]. Costic et al. [8] did not confirm the direction of testing, which prevents accurate comparison with Mazzocca et al. [18] and Deshmukh et al. [19] (Table 1). All three authors [8, 18, 19] implemented cyclic loading with similar loads, but the number of cycles varied. Mazzocca et al. [18] subjected the specimens to a constant load, which is probably less similar to physiological conditions than fluctuating loads. Mazzocca et al. [18] included the Weaver–Dunn procedure in their analysis, which was widely regarded as redundant at the time, so it may have been better to compare fewer techniques with larger sample sizes. Furthermore, Mazzocca et al. [18] did not establish normal results from all 42 specimens, but compared each technique to the normal conditions of the same 14 specimens. There seemed to be considerable differences between baseline results in the three groups of 14 specimens, but statistical analysis was not carried out to confirm this. There was variation in specimen age (Table 1), with cadavers in Mazzocca et al. [18] being 20 years older than those of Costic et al. [8]. Mazzocca et al. [18] found no difference between native conditions and the Weaver–Dunn technique, which contradicts the well-established principle that resection destabilises the joint [17], and may indicate an underlying error. However, it is less clear whether this principle applies in the absence of a compressive force. None of these reports assess stability in all three planes of motion and are limited by older CC reconstruction techniques.

**Table 1:** Summary of biomechanical testing of CC ligament reconstructions in cadaveric specimens

Study	Specimens	Mean age	Reconstruction technique	Directions assessed	Testing protocol
Costic et al. [8]	9	51.0 years	Anatomical CC ligament reconstruction with semitendinosus graft	Unconfirmed	100 cycles of loads fluctuating between 20 and 60 N, 1-h rest. Further 100 cycles of loads fluctuating between 20 and 90 N, then load-failure test
Deshmukh et al. [19]	21	64.7 years	Five non-anatomical CC reconstructions using different synthetic materials tested in 4 specimens each (one in 5)	Anterior, posterior and superior	1000 cycles of superior loading between 10 and 100 N, displacement of 1st and 1000th cycle recorded, then load-failure test
Mazzocca et al. [18]	42	72.8 years	Anatomical 2-bundle CC ligament (14 specimens) Single TightRope reconstruction of CC ligaments (14 specimens) Weaver and Dunn reconstruction (14 specimens)	Anterior, posterior and superior	10 cycles of 25 N, displacement in all three directions at 70 N, 3000 cycles of superior loading at 70 N, displacement testing in all three directions at 70 N, load-failure test

Newer reports demonstrate that TightRope systems can achieve normal stability in the superior direction [12]. However, the joint remains significantly less stable in the horizontal plane and for axial rotation. Walz et al. [20] reported that TightRope fixation had a higher load at failure and increased joint rigidity in comparison with native conditions. However, the AC ligaments were transected in their native control group, invalidating results.

Salzmann et al. [6] reported good clinical outcomes following CC reconstruction, with only one patient having mild residual pain, and no significant increase in CC distance at 30 months. However, seven of the initial 30 patients were excluded, three of whom had implant failure. Excluding those who experienced TightRope failure does not accurately represent the procedure. Furthermore, maintenance of reduction was expressed as an average and there were a number of over- and under-reductions which are not conveyed by this method. Thiel et al. [21] reported 100% satisfaction, but it appears that the one (of 12) who was not followed up experienced implant failure, corresponding with 91% (11/12) satisfaction. Reduction was lost in the initial two patients operated upon, as only a single TightRope device was implanted. Of the ten who had two devices implanted, nine retained complete reduction and one had failure. This is consistent with another study where a single TightRope technique demonstrated up to 50% failure rate [22]. Scheibel et al. [10] reported that only 57% (16/28) maintained reduction, but noticed that cases with sustained reduction had improved subjective and objective clinical outcomes. However, of the original 37 patients, only 28 attended final follow-up. The authors state that two declined due to being fully satisfied, and if this is true, may mean results appear to be poorer than they truly were. There is some evidence of coracoid avulsion fractures following TightRope implantation [23], but the patient in the relevant case report was poorly compliant with the post-operative regime.

Fewer techniques exist which reconstruct both the AC and CC ligament complexes. Lädermann et al. [24] described a technique in which suture bundles recreated the CC ligaments, with a distinct bundle encircling the anterior and posterior aspects of the joint, acting as the AC ligaments. Grutter and Peterson [7] reconstructed the CC ligaments with a tendon graft, which was reflected laterally to recreate the superior AC ligament. Mazzocca et al. [25] described a similar technique in which the graft reconstructions were reflected laterally to recreate the superior and posterior AC ligaments. Their procedure also contained 1 cm of distal clavicular excision, citing Weaver and Dunn’s [4] hypothesis of osteoarthritis prevention as justification. However, it has been demonstrated that AC ligament repair cannot offset the anteroposterior instability resulting from distal clavicle excision [26]. As the superior AC ligament and posterior capsule are the main stabilisers in the posterior plane [27], it is likely that an adapted Mazzocca et al. [25] procedure, omitting the clavicular excision, could be the superior technique described thus far.

Grutter and Peterson [7] found that their reconstruction had no significant difference in ultimate strength, and was only marginally less stiff, than the native ligaments. These authors loaded cadaveric specimens at 10 N/s along the axis of the conoid ligament until failure, but it is unlikely that the native complex is subjected to such a prolonged stress, and cyclical loading may have been a better representation. Furthermore, this method did not assess horizontal stability, which is the premise of AC ligament reconstruction. Saier et al. [28] demonstrated equivalent horizontal stability with native AC ligaments after 5000 cycles of horizontal loading.

Beitzel et al. [29] tested four AC reconstruction techniques and found that a technique resembling Mazzocca et al.’s [25] achieved horizontal stability comparable with the native complex. However, testing was limited to measuring displacement on application of a single 70-N load in the anterior and posterior directions, and it may be expected that motion would increase following repeated loading, due to stretching of the ligaments. These authors also noted that this technique inhibited physiological superior translations, but this was likely to be due to the CC complex component; the ligaments may be overly taut in the absence of cyclic loading. Beitzel et al. [29] also analysed rotational stability following cyclic loading and found that none of the techniques achieved physiological stability for axial rotation. However, the technique similar to Mazzocca et al.’s [25] achieved good results. Whilst results in other planes for these techniques may be unsatisfactory, it seems that AC ligament reconstruction can restore horizontal stability [28, 29].

It appears that only two papers have studied the clinical outcomes of procedures which reconstruct the AC and CC ligaments [30, 31]. Saccomanno et al. [30] used an autologous semitendinosus graft to recreate the conoid, trapezoid, superior AC and inferior AC ligaments. They performed this technique on a cohort of 18 patients with grade III AC dislocations. Saccomanno et al. [30] reported moderately good results, with 11% (2 of 18) experiencing asymptomatic recurrence of AC joint instability at a mean follow-up of 26 months [30]. The authors also noted that all patients had significantly improved DASH and Constant scores 26 months post-operatively compared to preoperatively [30]. Whilst this paper is useful in indicating the clinical feasibility of such procedures, it does not allow comparison with previous techniques to determine whether they achieve worse, equivalent or superior clinical and radiological results.

Tauber et al. [31] have performed the only study which prospectively compares the outcomes between a cohort with anatomical reconstruction and a cohort with sole CC ligament reconstruction. The anatomical reconstruction involved reconstruction of the trapezoid, conoid and superior AC ligaments with an autologous semitendinosus graft. This technique was referred to as triple-bundle reconstruction [31]. The second cohort received a single-bundle reconstruction, in which the tendon graft was passed through a single-bone tunnel, anchoring the clavicle to the coracoid process [31]. The two cohorts were obtained from two separate centres, with procedures carried out by two separate surgeons. The authors found that the anatomical reconstruction cohort had significantly better clinical and radiological outcomes. However, there were a number of uncontrolled variables which could also impact on these results. The CC ligament reconstruction was not constant between the triple-bundle and single-bundle reconstruction, and it seems unlikely that a fair comparison can be made between these techniques. Furthermore, distal clavicle resection was performed in the single-bundle cohort, but not the triple-bundle cohort. As previously described, resection of the distal clavicle has been shown to reduce horizontal stability and could have resulted in poorer outcomes. Also, there were differences in the demographics of the cohorts, with the single-bundle reconstruction cohort being significantly older, and having twice as many grade V injuries (although it was not clear whether the differences in grades of injury included reached significance).

## Discussion

Techniques which reconstruct the AC and CC ligaments achieve the best biomechanical results, but they are inferior to the normal joint (Table 2). This is the expected result, as the AC ligaments provide stability against horizontally directed forces, whilst the CC ligaments are the main resistors to vertical forces [32]. These techniques are still relatively new, and it may take a number of years for them to be refined. Although not yet described, there seems to be no reason why optimal CC ligament reconstructions cannot be adapted to include AC ligament reconstruction (Table 2). AC ligament reconstruction may be technically difficult due to their small size, but could potentially be performed arthroscopically, allowing assessment and treatment of concomitant glenoid cavity injuries.

**Table 2:** Comparison of the advantages and disadvantages of the three approaches in reconstructing the acromioclavicular joint: anatomical reduction; coracoclavicular ligament reconstruction; and anatomical reconstruction

Reconstruction approach	Advantages	Disadvantages
Anatomical reduction	Stability in vertical plane and to axial rotation equivalent to native complex [12]	Results in significantly less stability in horizontal plane [12] Requires two procedures for implantation and removal 81% maintained reduction at 1 year [3]
Coracoclavicular ligament reconstruction	Vertical stability equivalent to native complex [12] Single procedure Suggestion of good clinical outcomes for double TightRope technique, although data are limited [6, 21]	Results in significantly less stability in horizontal plane [12] Only three papers have investigated biomechanical outcomes, with limited ability for comparison [8, 18, 19] Various techniques described with little data comparing them
Anatomical reconstruction	Achieves overall biomechanical results most comparable to native complex [7] Only approach that restores horizontal stability [28] Promising early clinical outcomes [30] with some indication of improved results when compared with isolated CC reconstruction [31]	Techniques described thus far require further optimisation Current studies on clinical outcomes are inconclusive with the absence of data for long-term outcomes Increased technical difficulty

AC and CC ligament reconstruction techniques have achieved the most promising biomechanical results (Table 2). However, only two clinical studies have assessed their clinical feasibility. Whilst both studies portrayed promising results, they had a number of limitations which may impact the validity of these results. As such, it is not possible to make any conclusions as to whether anatomical reconstructions are the superior technique. Future clinical studies should compare anatomical reconstructions to isolated CC reconstructions in order to discern the optimal technique.

Assessing biomechanical properties in cadaveric specimens has a number of limitations. The advanced age of specimens is considerably different from the demographics of AC joint injury. Most authors isolated the joint through extensive soft tissue dissection and disarticulation of the glenohumeral and SC joints, resulting in a complex that almost certainly has altered biomechanical properties. It seems unlikely that direct force application to the joint would recreate physiological passive motion. Furthermore, specimens are often restricted to allow movement in only one plane, but motion in the native joint occurs in three planes simultaneously. Very few authors tested axial rotation, despite it being the most pronounced motion at the AC joint. The exception is Nüchtern et al. [12], who designed apparatus to allow three degrees of motion, and perhaps all future studies should recreate this method. Most authors discuss translations of the clavicle, but it is unclear whether this corresponds to rotations of the scapula and may better represent SC joint motions.

## Conclusion

Studies which have investigated the three surgical approaches to acute AC joint injury reconstruction have demonstrated that techniques which reconstruct both the AC and CC ligaments are biomechanically superior, but data on the clinical outcomes are lacking. Further studies are needed to explore more fully these surgical approaches to AC joint reconstruction in order to rectify this area of clinical uncertainty and ascertain whether there might be an optimal surgical solution.

## References

[1] Cooper ES (1861). New method of treating long-standing dislocations of the scapula-calvicular articulation. Am J Med Sci.

[2] Sage FP, Salvatore JE (1963). Injuries of the acromioclavicular joint: a study of results in 96 patients. South Med J.

[3] McKee M, Pelet S, McCormack RG, Harvey E, Papp S, Rouleau D, Furey A, Axelrod T, Buckley R, Guy P, Veillette C, Hall J, Wild L, Vicente M, Bedard L, Sinclair K, Reindl EHR, Berry G, Liew A, Gofton W, Kreder H, Hidy J, Cotu H, Stone T, Viskontas D, Boyer D, Moola F, Perey B, Lemke HM, Moon K, Zomar M, Reindl R, Houghton F, Foxall J, La Flamme Y, Beaumont P, Malo M, Parsons M, Kunz M, De Gorter R, Duffy P, Korley R, Puloski S, Carcary K, O’Brien PJ, Blachut PA, Broekhuyse HM, Lefaivre KA, Leung I, Azad T, Morison Z, Cote H, Blachut PA (2015). Multicentre randomized clinical trial of nonoperative versus operative treatment of acute acromioclavicular joint dislocation. J Orthop Trauma.

[4] Weaver JK, Dunn HK (1972). Treatment of acromioclavicular injuries, especially complete acromioclavicular separations. J Bone Joint Surg.

[5] Dimakopoulos P, Panagopoulos A, Syggelos SA, Panagiotopoulos E, Lambiris E (2006). Double-loop suture repair for acute acromioclavicular joint disruption. Am J Sports Med.

[6] Salzmann GM, Walz L, Buchmann S, Glabgly P, Venjakob A, Imhoff AB (2010). Arthroscopically assisted 2-bundle anatomical reduction of acute acromioclavicular joint separations. Am J Sports Med.

[7] Grutter PW, Peterson SA (2005). Anatomical acromioclavicular ligament reconstruction a biomechanical comparison of reconstructive techniques of the acromioclavicular joint. Am J Sports Med.

[8] Costic RS, Labriola JE, Rodosky MW, Debski RE (2004). Biomechanical rationale for development of anatomical reconstructions of coracoclavicular ligaments after complete acromioclavicular joint dislocations. Am J Sports Med.

[9] Blazar PE, Iannotti JP, Williams GR (1998). Anteroposterior instability of the distal clavicle after distal clavicle resection. Clin Orthop Relat Res.

[10] Scheibel M, Dröschel S, Gerhardt C, Kraus N (2011). Arthroscopically assisted stabilization of acute high-grade acromioclavicular joint separation. Am J Sports Med.

[11] McConnell AJ, Yoo DJ, Zdero R, Schemitsch EH, McKee MD (2007). Methods of operative fixation of the acromio-clavicular joint: a biomechanical comparison. J Orthop Trauma.

[12] Nüchtern JV, Sellenschloh K, Bishop N, Jauch S, Briem D, Hoffmann M, Lehmann W, Pueschel K, Morlock MM, Rueger JM, Groβterlinden LG (2013). Biomechanical evaluation of 3 stabilization methods of acromioclavicular joint dislocations. Am J Sports Med.

[13] Di Francesco A, Zoccali C, Colafarina O, Pizzoferrato R, Flamini S (2012). The use of hook plate in type III and V acromioclavicular Rockwood dislocations: clinical and radiological midterm results and MRI evaluation in 42 patients. Injury.

[14] Yoo YS, Seo YJ, Noh KC, Patro BP, Kim DY (2011). Arthroscopically assisted anatomical coracoclavicular ligament reconstruction using tendon graft. Int Orthop.

[15] Sim E, Schwarz N, Höcker K, Berzlanovich A (1995). Repair of complete acromioclavicular separations using the acromioclavicular-hook plate. Clin Orthop Relat Res.

[16] Harris RI, Wallace AL, Harper GD, Goldberg JA, Sonnabend DH, Walsh WR (2000). Structural properties of the intact and the reconstructed coracoclavicular ligament complex. Am J Sports Med.

[17] Costic RS, Jari R, Rodosky MW, Debski RE (2003). Joint compression alters the kinematics and loading patterns of the intact and capsule-transected AC joint. J Orthop Res.

[18] Mazzocca AD, Santangelo SA, Johnson ST, Rios CG, Dumonski ML, Arciero RA (2006). A biomechanical evaluation of an anatomical coracoclavicular ligament reconstruction. Am J Sports Med.

[19] Deshmukh AV, Wilson DR, Zilberfarb JL, Perlmutter GS (2004). Stability of acromioclavicular joint reconstruction: biomechanical testing of various surgical techniques in a cadaveric model. Am J Sports Med.

[20] Walz L, Salzmann GM, Fabbro T, Eichhorn S, Imhoff AB (2008). The anatomic reconstruction of acromioclavicular joint dislocations using 2 tightrope devices. Am J Sports Med.

[21] Thiel E, Mutnal A, Gilot GJ (2011). Surgical outcome following arthroscopic fixation of the acromioclavicular joint disruption with the tightrope device. Orthopedics.

[22] Lim YW, Sood A, van Riet RP, Bain GI (2007). Acromioclavicular joint reduction, repair and reconstruction using metallic buttons—early results and complications. Tech Shoulder Elbow Surg.

[23] Bindra J, van Den Bogaerde J, Hunter JC (2011). Coracoid fracture with recurrent AC joint separation after tightrope repair of AC joint dislocation. Radiol Case Rep.

[24] Lädermann A, Grosclaude M, Lübbeke A, Christofilopoulos P, Stern R, Rod T, Hoffmeyer P (2011). Acromioclavicular and coracoclavicular cerclage reconstruction for acute acromioclavicular joint dislocations. J Shoulder Elbow Surg.

[25] Mazzocca AD, Conway JE, Johnson S, Rios CG, Dumonski ML, Santangelo SA, Arciero RA (2004). The anatomic coracoclavicular ligament reconstruction. Oper Tech Sports Med.

[26] Corteen DP, Teitge RA (2005). Stabilization of the clavicle after distal resection a biomechanical study. Am J Sports Med.

[27] Klimkiewicz JJ, Williams GR, Sher JS, Karduna A, Des Jardins JD, Iannotti JP (1999). The acromioclavicular capsule as a restraint to posterior translation of the clavicle: a biomechanical analysis. J Shoulder Elbow Surg.

[28] Saier T, Venjakob AJ, Minzlaft P, Föhr P, Lindell F, Imhoff AB, Vogt S, Braun A (2015). Value of additional acromioclavicular cerclage for horizontal stability in complete acromioclavicular separation: a biomechanical study. Knee Surg Sports Traumatol Arthrosc.

[29] Beitzel K, Obopilwe E, Apostolakos J, Cote MP, Russell RP, Charette R, Singh H, Arciero RA, Imhoff AB, Mazzocca AD (2014). Rotational and translational stability of different methods for direct acromioclavicular ligament repair in anatomic acromioclavicular joint reconstructions. Am J Sports Med.

[30] Saccomanno MF, Fodale M, Capasso L, Cazzato G, Milano G (2014). Reconstruction of the coracoclavicular and acromioclavicular ligaments with semitendinosus tendon graft: a pilot study. Joints.

[31] Tauber M, Valler D, Lichtenburg S, Magosch P, Moroder P, Habermeyer P (2016). Arthroscopic stabilization of chronic acromioclavicular joint dislocations triple-versus single-bundle reconstruction. Am J Sports Med.

[32] Oki S, Matsumura N, Iwamoto W, Ikegami H, Kiriyama Y, Nakamura T, Toyama Y, Nagura T (2012). The function of the acromioclavicular and coracoclavicular ligaments in shoulder motion: a whole cadaver study. Am J Sports Med.

